# Folate and Other B Vitamins in Brain Health and Disease

**DOI:** 10.3390/nu15112525

**Published:** 2023-05-29

**Authors:** Edward V. Quadros

**Affiliations:** Department of Medicine, SUNY Downstate Medical Center, 450 Clarkson Avenue, Brooklyn, NY 11209, USA; edward.quadros@downstate.edu; Tel.: +1-718-270-4203

B vitamins as a group play essential roles in a multitude of metabolic reactions involved in cellular replication, energy production, the synthesis of intermediary compounds, and neurotransmitters. Therefore, an abundant and constant supply of these micronutrients is needed for reproductive health, pregnancy, fetal and neonatal development, and brain function and health throughout life. The need for these nutrients becomes especially critical in later life when the nutrient intake and absorption may be compromised. The need for these vitamins and their role in maintaining normal health, especially brain function, is the topic of this Special Issue of *Nutrients* entitled “*Folate and Other B Vitamins in Brain Health and Disease*”. Original articles and reviews covering these topics will be presented in two volumes. The journal is now soliciting new articles and reviews for volume two of this Special Issue. The first volume is now complete and covers the topics outlined below.

The first original article reports on vitamin B complex and experimental autoimmune encephalomyelitis as well as the role of these vitamins in the attenuation of the clinical signs and gut microbiota dysbiosis [1]. This study investigated the neuroprotective effects of B-complex vitamins in a rat model of experimental autoimmune encephalomyelitis and found beneficial effects in attenuated clinical signs including a reduced duration of disease, thereby contributing to a faster recovery. A significant improvement in nerve and muscle pathology, the immune response, and gut microbiota was also observed.

The second report identifies a connection between the dietary intake composition of choline and betaine and a reduced severity of visceral obesity-related hepatic steatosis [2]. In a case–control study of 105 patients with hepatic steatosis, the methyl-donor nutrient (MDNs: choline, betaine, and folate) intake was assessed using a validated quantitative food frequency questionnaire. After adjustment for multiple risk factors, the total choline intake was the most significant dietary determinant of hepatic steatosis in patients with visceral obesity and non-alcoholic fatty liver disease. A low intake of choline and betaine predicted increased visceral-obesity-related hepatic steatosis. A combined high intake of choline and betaine, but not folate, was associated with an 81% reduction in visceral-obesity-associated hepatic steatosis.

Food fortification and increased vitamin intake have led to higher folic acid (FA) consumption by pregnant women, and some studies have suggested that buildup of unmetabolized folic acid may have negative effects on folate pathways. In a pregnant mouse model utilizing a folic-acid-supplemented diet, a fivefold-higher FA than the recommended level led to hyperactivity-like behavior and memory impairment in pups [3]. Disturbed choline/methyl metabolism and altered placental gene expression were identified. The study examined two developmental stages, postnatal day 30 and embryonic day 17.5, and observed sex-specific transcription changes in the P30 cerebral cortex and E17.5 cerebrum, with changes in genes involved in neurotransmission, neuronal growth and development, and angiogenesis. This study provides insight into potential deleterious consequences of folate over-supplementation during pregnancy. 

Dietary folate is mostly converted to 5-Methyltetrahydrofolate (5-MTHF) during absorption and accounts for most of the folate in circulation. Therefore, measuring this form provides an accurate estimate of the folate status. The next study reports on developing a sensitive platform to measure methylfolate in subjects with MTHFR and PON1 gene polymorphisms [4]. An inadequate level of 5-MTHF along with the T variant of MTHFR C677T have been suggested to be associated with an increased risk of developing mental illness, whereas the PON1 SNP variant provides a protective role. Inadequate MTHF production and recycling of folate has been linked to these disorders. However, reports validating the methodology for plasma 5-MTHF levels in schizophrenia patients are limited. A sensitive LC–MS/MS system using an amide column and calibration curve was applied to schizophrenia patients and healthy controls in Taiwan, and the differences between the subgroups were discussed. The mean plasma 5-MTHF levels in schizophrenia patients were lower than those in the healthy controls. The 5-MTHF concentrations were significantly lower in male carriers than in female carriers, especially in subjects who were MTHFR CT/PON1 Q allele carriers. This quantitative system, which employed sensitive and simple processing methods, was successfully applied, and it was identified that schizophrenic patients had significantly lower levels of 5-MTHF. Lower plasma 5-MTHF with lower concentrations was observed in male subjects.

Folate (B9) is essential for reproductive health, pregnancy, and normal fetal development. Folic acid, a synthetic form of folate, is widely used in multivitamin preparations, food fortification, and as a supplement during pregnancy. At physiologic dosing, this vitamin is rapidly converted to methylfolate and absorbed. However, at higher doses, some unconverted vitamins may be absorbed. Recent studies have suggested that unconverted folic acid may affect folate pathways by interfering with folate-dependent enzymes and metabolism. Folate deficiency during pregnancy has been associated with developmental abnormalities such as neural tube defects in the fetus and autism spectrum disorders in children. These disorders can be prevented and treated with high-dose vitamin B9. Folate receptor antibodies are significantly associated with the disruption of brain development in the fetus and function in later life. Folate is actively transported to the fetus, and this transport can be blocked by an antibody against the folate receptor. Folate receptor antibodies are significantly associated with neural tube pregnancy and autism spectrum disorders, and folinic acid treatment in the latter condition has shown improvement in core behavioral deficits along with normalizing the CSF folate status.

Food fortification and folic acid supplementation during pregnancy have been implemented as strategies to prevent fetal malformations during pregnancy. However, with the emergence of conditions wherein folate metabolism and transport are disrupted, such as folate receptor alpha autoantibody (FRαAb)-induced folate deficiency, it is critical to find a folate form that is safe and effective for pharmacologic dosing for prolonged periods. The study in rats evaluated absorption and tissue distribution of folate forms to identify a specific folate form suitable for supplementation during pregnancy [5]. The study examined absorption and tissue distribution of folic acid (PGA), 5-methyl-tetrahydrofolate (MTHF), l-folinic acid (levofolinate), and d,l-folinic acid (Leucovorin) in rats and showed that all forms are converted to MTHF while some unconverted folate forms are transported into the blood, especially PGA. The study identified the rapid distribution of absorbed folate to the placenta and fetus. FRαAb also accumulates rapidly in the placenta and blocks folate transport to the fetus, and high folate concentrations are needed to circumvent or overcome the blocking of FRα. In the presence of FRαAb, both Leucovorin and levofolinate are absorbed and distributed to tissues better than the other forms. However, only 50% of the leucovorin is metabolically active, whereas levofolinate is fully active and generates higher tetrahydrofolate (THF). The study suggests that levofolinate should be the folate form of choice during pregnancy and for other disorders where large daily doses of folate are needed, as well as in food fortification. In a subsequent study, the authors examined the brain’s uptake of folate forms in the presence of folate receptor alpha antibodies (FRαAb) [6]. In young rats, FRαAb injected intraperitoneally (IP) localizes to the choroid plexus and blood vessels including the capillaries throughout the brain parenchyma. Biotin-tagged folic acid shows distribution in the white matter tracts in the cerebrum and cerebellum. Three forms of folate, namely folic acid, D,L-folinic acid, and levofolinate, when orally administered, are converted to methylfolate while L-methylfolate is absorbed, and all are efficiently distributed to the brain. However, significantly higher folate concentration is seen in the cerebrum and cerebellum with levofolinate in the presence or absence of FRαAb. These results in the rat model support testing levofolinate to treat children with ASD.

The review included in this issue discusses cerebral folate deficiency syndrome and suggests strategies for early diagnosis and treatment [7]. Cerebral folate deficiency syndrome (CFDS) is defined as any neuropsychiatric or developmental disorder characterized by decreased CSF folate levels in the presence of normal folate status outside the nervous system. The specific clinical profile appears to be largely determined by the presence or absence of intrauterine folate deficiency as well as the postnatal age at which cerebral folate deficiency occurs. The primary cause of CFDS is identified as the presence of serum folate receptor alpha (FRα) autoantibodies impairing folate transport across the choroid plexus to the brain, whereas, in a minority of cases, mitochondrial disorders, inborn errors of metabolism, and loss-of-function mutations of the FRα (*FOLR1*) gene are identified. Early recognition and diagnosis of CFDS and prompt intervention are important to improve the prognosis with successful outcomes. This article focuses on FRα autoimmunity and its different age-dependent clinical syndromes, the diagnostic criteria, and treatments to be considered, including prevention strategies in this at-risk population that includes ASD. The two volumes of this Special Issue will cover current new research and clinical studies, with emphasis on the role of B vitamins in brain development and function and in the ageing brain.
